# Diagnostic role of fluorodeoxyglucose positron emission tomography-computed tomography in prostate cancer

**DOI:** 10.3892/ol.2014.1997

**Published:** 2014-03-24

**Authors:** YIYAN LIU

**Affiliations:** Nuclear Medicine Service, Department of Radiology, New Jersey Medical School, Newark, NJ 07103, USA

**Keywords:** fluorine-18 fluorodeoxyglucose positron emission tomography-computed tomography, metastasis, prostate cancer, prostate-specific antigen

## Abstract

The role of fluorine-18 fluorodeoxyglucose (FDG) positron emission tomography (PET)-computed tomography (CT) in prostate cancer remains controversial due to a limited number of previous clinical investigations. The aim of the present retrospective study was to assess the diagnostic value of FDG PET-CT in prostate cancer, with an emphasis on the detection of metastatic disease. Twenty-five relevant cases of patients with newly diagnosed prostate cancer, referred for staging, or with a history of prostate cancer or recent prostate specific antigen (PSA) relapse, referred for the detection of metastatic disease, were included in the present study. None of the patients had known imaging or pathological evidence of metastatic disease prior to FDG PET-CT, however, the PSA levels had been recorded in all patients in the two months prior to FDG PET-CT imaging. Verification of the FDG PET-CT observations was made by biopsy, regional diagnostic CT and/or whole-body bone scintigraphy. The sensitivity of FDG PET-CT in identifying untreated primary lesions was only 33% (3/9). However, FDG PET-CT detected metastatic disease in six of the nine patients who underwent initial staging. Out of 16 patients with previous treatments and recent PSA relapse, FDG PET-CT successfully identified metastatic diseases in 12 and tumor recurrence within the prostatic fossa of two patients. The difference in the PSA levels was identified to be statistically significant between the FDG PET-CT-positive and -negative subgroups of the 16 restaging patients. The results indicated that FDG PET-CT is not useful for the diagnosis of prostate cancer, but may aid with the detection of metastatic disease in appropriately selected patients.

## Introduction

Prostate cancer is known clinically as a heterogeneous disease, characterized by biological behavior that ranges between indolent and aggressive states. Although the initial diagnosis of prostate cancer is relatively straightforward, as it is based on prostate-specific antigen (PSA) screening and confirmatory biopsy, the accurate staging and detection of recurrent and metastatic disease remains clinically challenging. Post-therapeutic biochemical failure in prostate cancer represents a diagnostic dilemma to urologists, oncologists and radiologists.

Fluorine-18 fluorodeoxyglucose (FDG) positron emission tomography (PET)-computed tomography (CT) has been widely used for diagnosis, initial staging, restaging, monitoring of therapeutic response and prognostication in various types of cancer, however, its role in prostate cancer remains controversial. The small number of previously published observations indicated that unlike the majority of malignancies, prostate tumors are characterized by slow glycolysis and low FDG-avidity on PET images (1,2). Significant overlap has been identified between FDG uptake in prostate cancer and benign prostate hyperplasia. An additional confounding problem is that FDG is normally excreted by the kidneys and intense activity in the distended urinary bladder usually obscures the prostate and interferes with the identification of pelvic lymph nodes (1–4). Therefore, application of FDG PET-CT in prostate cancer is generally discouraged.

The purpose of the present retrospective study was to evaluate the diagnostic value of FDG PET-CT in prostate cancer, with an emphasis on the detection of metastatic disease.

## Patients and methods

### Ethics

The present retrospective study was approved by the Institutional Review board of the University of Medicine and Dentistry of New Jersey (Newark, NJ, USA). The relevant cases were identified and selected from a computerized database of patients who underwent FDG PET-CT imaging at the Advanced Imaging Center, University Hospital (Newark, NJ, USA) between January 2006 and December 2011. The medical records were retrospectively reviewed for pathological, therapeutic and radiological information.

### Patients

In total, 25 relevant cases of patients with newly diagnosed prostate cancer, referred for staging (nine cases), or with a history of prostate cancer or recent PSA relapse, referred for detection of metastatic disease (16 cases), were included. None of the patients had known imaging or pathological evidence of metastatic disease prior to FDG PET-CT, however, the PSA levels had been recorded in all patients within two months prior to FDG PET-CT imaging. In total, 12 patients with imaging that suggested metastatic disease prior to FDG PET-CT were excluded from the analysis. An additional 15 cases were excluded from the study for the following reasons: i) No data were available at the time of FDG PET-CT imaging, including Gleason score at diagnosis, detailed therapeutic history or PSA levels prior to FDG PET-CT; ii) no further confirmatory tests were available following a positive FDG PET-CT; and iii) cases with concurrent prostate cancer and other malignancies, for instance, an additional tumor other than prostate cancer, such as lung or colon cancer, or lymphoma, were referred for FDG PET-CT.

### Combined FDG PET-CT

Combined FDG PET-CT was performed using a PET-CT scanner (Discovery LS; GE Healthcare, Amersham, UK) and standard techniques. The maximum standardized uptake value (SUV_max_) of lesions was recorded. The definition of a positive scan was that the observations were consistent with that of a malignant or metastatic disease. By contrast, a negative scan indicated no apparent abnormalities and the observations were not indicative of a malignancy. The interpretation criteria for the positive FDG PET-CT imaging were as follows: i) Focal uptake with an SUV_max_ of >3.0 in the prostatic fossa; ii) nodal SUV_max_ of >3.0 regardless of its size; iii) sclerotic or lytic osseous lesion with an SUV_max_ of >3.0; and iv) other CT identified and FDG-avid lesions with an SUV_max_ of >3.0. Non-FDG-avid lymph nodes and bone lesions were interpreted as negative.

### Verification of FDG PET-CT observations

The nine patients for initial staging all underwent prostate biopsy and were diagnosed with cancer prior to FDG PET-CT. In addition, one patient in the restaging group underwent prostate biopsy following a positive imaging observation in the prostatic fossa. For metastatic lesions that were identified by FDG PET-CT, confirmatory biopsies were performed in three cases (lymph node dissection, lung lesion resection and bone biopsy). All other patients underwent further verification imaging studies within the following three months after positive or negative FDG PET-CT studies, with the exception of one patient with negative FDG PET-CT in the restaging group. For the initial staging and restaging cases, regional diagnostic CT (predominantly abdomen and pelvis) was performed if the FDG PET-CT indicated nodal metastasis. Regional diagnostic CT and/or whole-body bone scintigraphy were performed if FDG PET-CT indicated bone metastasis and diagnostic CT of the abdomen and pelvis was performed if FDG PET-CT was negative for metastatic disease.

### Statistical analysis

A paired t-test was used to perform the for statistical analyses. P<0.05 was considered to indicate a statistically significant difference.

## Results

### Patient characteristics

Table I summarizes the characteristics of 25 eligible patients, including PSA level, Gleason score, FDG PET-CT observations and confirmatory or verification methods. The patients were divided into two groups, initial staging and restaging.

### FDG PET-CT for initial staging of prostate cancer

Nine patients were in the initial staging group, with a mean age of 61±9 years (range, 50–73 years) and a mean PSA level of 291±363 ng/ml (range, 6.1–980 ng/ml). All patients had been newly diagnosed with prostate cancer by prostatic biopsy. FDG PET-CT demonstrated abnormal uptake of the prostate in only three cases and no abnormal prostate uptake was observed in the remaining six cases, including two cases with PSA levels of 511 and 369 ng/ml. However, FDG PET-CT successfully detected metastatic lesions in the lymph nodes and/or bones of six cases.

### FDG PET-CT for patients with post-therapeutic biochemical recurrence

In total, 16 patients were included in the restaging group with a mean age of 66±6 years (range, 55–72 years) and a mean PSA level of 99±225 ng/ml (range, 3.1–680 ng/ml). Eight patients had undergone radical prostatectomy and eight had received prostate-preserving treatments, such as brachytherapy, hormonal therapy or hormone combined with radiation therapy. FDG PET-CT only detected two recurrences within the prostate and recurrence in one patient was confirmed by prostatic biopsy. By contrast, FDG PET-CT identified metastatic diseases in 12 patients, which were confirmed by biopsy, diagnostic CT and/or bone scintigraphy in the subsequent three months. Of these 12 patients, six exhibited bone lesions, three exhibited nodal lesions, two exhibited bone and nodal diseases, and one exhibited lung metastasis. FDG PET-CT failed to detect an abnormality in three patients, of which two were found to exhibit enlarged pelvic nodes on the follow-up CT one month later; one patient (patient 22) did not undergo further imaging.

### Role of FDG PET-CT in prostate cancer

**Figure 1:**
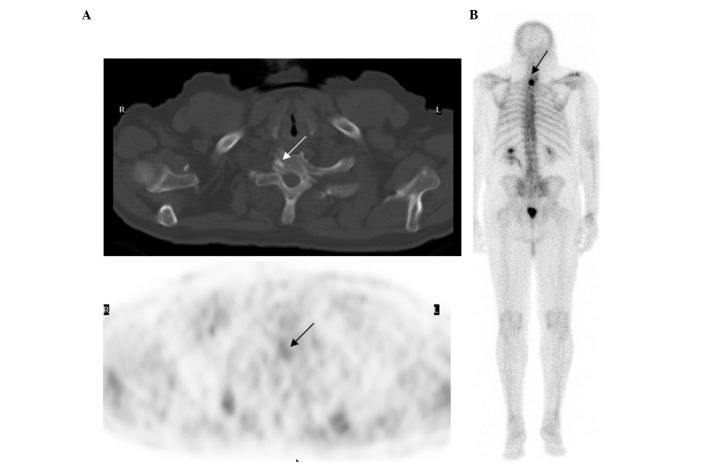
(A) Transaxial fluorine-18 fluorodeoxyglucose (FDG) positron emission tomography (PET)-computed tomography (CT) images of the upper chest of patient 13. The 70-year-old male exhibited a prostate-specific antigen relapse (PSA level, 67 ng/ml) five years following radical prostatectomy. The first bone scintigraphy and pelvic CT were obtained one month prior to the FDG PET-CT and the two were negative. FDG PET-CT demonstrated a small sclerotic density with mild uptake (SUV_max_, 3.5), which indicated metastasis in the right-side of the T1 vertebral body (indicated by the arrows). (B) Repeat bone scintigraphy one month later confirmed the FDG PET-CT-identified lesion (indicated by the arrow).

**Figure 2:**
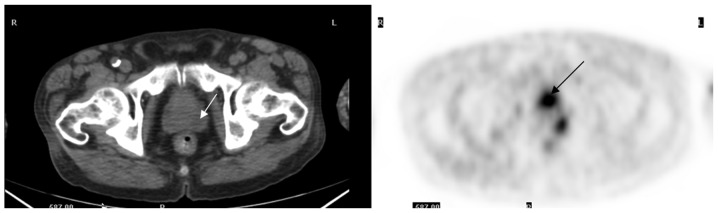
Transaxial fluorine-18 fluorodeoxyglucose (FDG) positron emission tomography (PET)-computed tomography (CT) images obtained from a 72-year-old male (patient 21) following external beam radiotherapy for prostate cancer. The patient’s series prostate-specific antigen (PSA) levels were unremarkable until two years later when the PSA level was 7.8 ng/ml. FDG PET-CT demonstrated an FDG-avid lesion in the left-side of the prostate (indicated by the arrows), which was confirmed as recurrent adenocarcinoma by transrectal biopsy.

In nine newly diagnosed prostate cancer patients, the sensitivity of FDG PET-CT in identifying untreated primary lesions was only 33% (3/9). However, FDG PET-CT detected metastatic disease in six of the nine patients who underwent initial staging. Out of 16 patients with previous treatments and recent PSA relapse, FDG PET-CT successfully identified metastatic diseases in 12 and tumor recurrence within the prostatic fossa of two patients, one of which exhibited prostate cancer recurrence and metastasis. The positive rate of FDG PET-CT was 81% (13/16). Fig. 1 shows an example of the role of FDG PET-CT in the detection of metastatic disease in prostate cancer. Five years following radical prostatectomy, a 70-year-old male (patient 13) exhibited a PSA relapse of 67 ng/ml. The first bone scintigraphy and pelvic CT were obtained for the initial workups one month prior to FDG PET-CT and the two were negative. FDG PET-CT demonstrated a small sclerotic density with mild uptake (SUV_max_, 3.5) that indicated metastasis in the right-side of the T1 vertebral body. Repeat bone scintigraphy that was conducted one month later confirmed the FDG PET-CT-identified lesion. Fig. 2 shows transaxial FDG PET-CT images obtained from a 72-year-old male post external beam radiotherapy for prostate cancer (patient 21). The patient’s series PSA levels were unremarkable until two years later when the PSA level was 7.8 ng/ml. FDG PET-CT demonstrated an FDG-avid lesion in the left-side of the prostate, which was confirmed as recurrent adenocarcinoma via a transrectal biopsy.

### Correlation between PSA levels and positive FDG PET-CT studies

**Figure 3:**
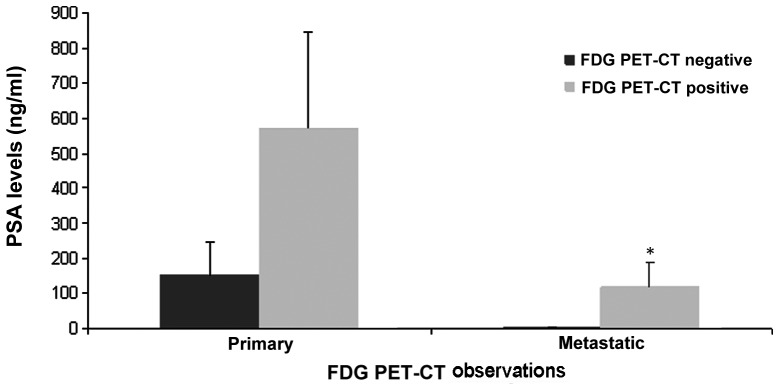
Mean PSA levels in the subgroups of patients. For primary prostate tumors in the initial staging group, the mean PSA level was 153±92 ng/ml in the FDG PET-CT-negative cases and 570±274 ng/ml in the FDG PET-CT-positive cases; the difference in the PSA levels was not identified to be statistically significant (P>0.05). The mean PSA levels in the restaging (metastatic) group were 4.7±0.49 ng/ml in the FDG PET-CT-negative cases and 120±68 ng/ml in the the FDG PET-CT-positive cases. The difference in the PSA levels was identified to be statistically significant (^*^P<0.05) between the two subgroups of restaging patients. FDG, fluorine-18 fluorodeoxyglucose; PET-CT, positron emission tomography-computed tomography; PSA, prostate-specific antigen.

Fig. 3 represents the mean PSA levels in all subgroups of the patients. For primary prostate tumors in the initial staging group, the mean PSA levels were 153±92 ng/ml in the FDG PET-CT-negative cases and 570±274 ng/ml in the FDG PET-CT-positive cases. Statistical analysis identified no significant difference in the initial serum PSA levels between patients with positive and negative FDG PET-CT for primary prostate lesions. The reason for this insignificant difference may be due to the wide variation in the range of PSA levels and the small case number. However, no abnormal uptake was identified in the prostate of the two patients with PSA levels of 369 and 511 ng/ml.

The mean PSA levels in the restaging (metastatic) group were 4.7±0.49 ng/ml in patients with negative FDG PET-CT scans (three cases) and 120±68 ng/ml in patients with positive FDG PET-CT scans (13 cases). The difference in the PSA levels was identified to be statistically significant (P<0.05) between the two subgroups of the restaging patients.

## Discussion

FDG is a non-physiological compound with a chemical structure extremely similar to that of naturally occurring glucose. Similarly to glucose, FDG enters the cells through membrane glucose transporter proteins, which are commonly overexpressed in cancer cells (5). FDG is actively transported into the cell through the membrane glucose transporters and converted into FDG-6-phosphate by hexokinase. Since FDG-6-phosphate is not a substrate for the enzyme responsible for the next step in glycolysis, it is then trapped and accumulates in the cell in proportion to its glucose metabolic activity. Malignant cells exhibit increased FDG accumulation due to increased membrane transporters, increased intracellular hexokinase and low levels of glucose-6-phosphatase (5,6).

A limited number of previous studies demonstrated a low sensitivity of FDG PET-CT in the detection of primary prostate cancer, indicating that FDG PET-CT may not be useful in the diagnosis or staging of clinically organ-confined disease (7). Previously, Oyama *et al* (8) reported a 64% sensitivity of FDG PET-CT in detecting primary prostate cancer; however, the subjects in the study exhibited high serum PSA levels (mean PSA level, 251 ng/ml), as well as advanced stage and aggressive cancer. Liu *et al* (9) previously reported that FDG PET-CT only exhibited 4% sensitivity in detecting prostate cancer among 24 patients, with a mean serum PSA level of 13.6 ng/ml. In addition, Minamimoto *et al* (10) reported a 51.9% sensitivity of FDG PET-CT in 50 subjects with increasing PSA levels that were suspected of having prostate cancer. The results showed that FDG PET-CT was appropriate for detecting peripheral zone prostate cancer in patients that were considered to exhibit more than an intermediate risk. The results of the current study showed only 33% sensitivity of FDG PET-CT for identifying primary prostate lesions in newly diagnosed patients, which is consistent with the previously described observations. Although high PSA levels are likely to increase the possibility of FDG-avidity of primary tumors, no statistical difference was identified between the two subgroups of patients in the current series due to the small case number.

The low sensitivity of FDG PET-CT in identifying prostate cancer is considered to be predominantly due to the slow rate of glycolysis of tumor cells as a result of relatively slow tumor growth. Previous laboratory studies have revealed that glucose transporter mRNA and protein are only weakly expressed in human prostate cancer tissues, which is considered to account for its low FDG-avidity (11). A previous *in vitro* study also indicated that glucose may not be required for androgen-dependent prostate cancer cells since LNCaP cells grow at controlled rates even in a medium containing only 0.05 g/l glucose (12). In addition, Liu *et al* (13) reported that all benign and malignant prostate cells are characterized by a dominant uptake of fatty acid compared with glucose.

Information on lymph node status is important when planning appropriate treatment for patients with newly diagnosed prostate cancer. Although conventional imaging modalities, such as CT and magnetic resonance imaging (MRI), are often used to detect nodal disease, previous observations have indicated that FDG PET-CT is more sensitive than anatomic imaging in the detection of nodal metastasis. Heicappell *et al* (14) investigated the use of FDG PET in determining pelvic lymph node metastases and found that FDG PET was positive in four of the six patients with histologically confirmed lymph node spread, which was a superior outcome compared with the CT imaging. In the present study, FDG PET-CT identified five cases with nodal disease in the nine patients that were newly diagnosed with prostate cancer, including two cases with distant nodal lesions in the mediastinum and neck. In addition, five patients were found to exhibit osseous metastasis on FDG PET-CT. It is clear that FDG PET-CT has an advantage, with regards to whole-body data acquisition and ability to detect more distant or unexpected lesions, compared with a regional diagnostic CT or MRI.

Post-therapeutic biochemical failure in prostate cancer represents a diagnostic dilemma and poses a great challenge to urologists and oncologists. FDG PET-CT has shown a promising role in the detection of local recurrence or metastatic disease. In a previous study of 24 patients with rising PSA levels following treatment for localized prostate cancer, CT and FDG PET-CT were obtained prior to pelvic lymph node dissection (15). The CT was negative in all cases, whereas FDG PET-CT detected 75% of histopathologically proven metastases. The sensitivity, specificity, accuracy and positive and negative predictive values of FDG PET-CT in detecting metastatic pelvic lymph nodes were 75*,* 100, 83, 100 and 68%, respectively. In an additional retrospective study of 91 patients with PSA relapse following prostatectomy, and validation of tumor presence by biopsy or clinical and imaging follow-up, FDG PET-CT detected local or systemic disease in 31% of patients (16). In the current case series, FDG PET-CT detected metastatic diseases and less frequently, recurrent prostate lesions, in 81% (13/16) of patients, which significantly contributed to the patient management. The majority of metastases were identified in the lymph nodes and bone. The sites of osseous metastases were randomly distributed, the most common being the spine and pelvis. In addition, the majority of lymph node lesions were located in the pelvis and lower abdomen, although nodal lesions in a few cases were detected in the upper torso, such as the mediastinum or neck. The results demonstrated a promising role of FDG PET-CT in the detection of metastatic disease. Clearly, the present results showed a higher positive rate of FDG PET-CT to detect metastatic disease than that which was previously reported (16). The reason of this difference is likely to be secondary to the selection of the patients; mean PSA levels were 99±225 ng/ml in the current study, but only 11±10 ng/ml in the previously published cases (16).

The present study demonstrated that FDG PET-CT has a much higher sensitivity for the detection of metastatic lesions than for primary prostate cancer, which may result from the progression of the energetic metabolism in metastatic lesions compared with the primary lesion; increased cell proliferation and accelerated glucose metabolism. A previous *in vitro* study indicated that the level of expression of glucose transporter 1 was higher in the poorly differentiated cell lines, DU-145 and PC-3, than in the well-differentiated hormone-sensitive LNCaP cell line, demonstrating that glucose metabolism increases with the progression of malignancy (17).

While PSA levels are not definitely associated with positive FDG PET-CT observations for primary prostate lesions, high PSA levels in restaging may predict a positive FDG PET-CT scan for metastatic and/or recurrent disease. In the restaging group, all three patients with negative FDG PET-CT exhibited low PSA levels (4.7±0.49 ng/ml), whereas the patients with positive scans exhibited much higher PSA levels (mean, 120±68 ng/ml) with only three cases with a PSA of <10 ng/ml.

In recent years, much effort has been made to develop novel radiotracers for PET imaging of prostate cancer, such as tracers that identify cell membrane turnover, protein synthesis, DNA synthesis and testosterone metabolism within the prostate, however, they each have certain limitations in clinical applications (18–20). As the only readily available PET tracer on the market worldwide, the role of FDG in PET imaging of prostate cancer cannot be ignored purely based on the limited number of previous negative observations. FDG PET-CT is useful for the detection of metastatic disease in prostate cancer in certain patient groups, such as those with high PSA levels.

The present study was retrospective and a major limitation was referral bias. Since FDG PET-CT is not a routine or standard imaging modality in prostate cancer, only patients with a high pretest possibility of metastasis were referred for FDG PET-CT by the urologist or oncologist, which may account for the high PSA levels and high positive rate of metastatic disease in the patients of the present study. In addition, following the exclusion of cases without detailed historical information, particularly PSA levels two months prior to FDG PET-CT imaging, the total case number was small, which limited the statistical power of the data.

In conclusion, FDG PET-CT may impact the clinical management of patients with prostate cancer, although this impact may be lower than that for other cancers. Although FDG PET-CT has a low sensitivity for identifying primary prostate lesions and is not useful for the diagnosis of prostate cancer, it may aid in the detection of metastatic disease in appropriately selected patients. In patients with post-therapeutic PSA relapse, FDG PET-CT has a clear role in the detection of metastatic lesions, particularly in those with high PSA levels. Furthermore, FDG PET-CT has the advantage of whole-body data acquisition and the ability to detect more distant or unexpected lesions compared with a regional diagnostic CT or MRI.

## Figures and Tables

**Table I:** Patient characteristics and FDG PET-CT observations.

					FDG PET-CT observation			
						
Patient no.	Age, years	Gleason score	Therapeutic history	PSA on imaging, ng/ml	Prostatic fossa	Lymph nodes	Bone (or other)	Confirmatory test
1	59	7	Initial staging	6.8	−	+	+	BS and CT
2	61	9	Initial staging	14	−	+	−	CT and PLND
3	52	6	Initial staging	49	+	−	−	CT and BS
4	50	7	Initial staging	6.1	−	−	−	CT and BS
5	73	8	Initial staging	680	+	+	+	CT and BS
6	69	6	Initial staging	9.6	−	−	−	CT
7	53	8	Initial staging	511	−	+	+	BS and CT
8	59	7	Initial staging	369	−	−	+	BS and CT
9	71	9	Initial staging	980	+	+	+	BS and CT
10	64	6	Prostatectomy	3.1	−	−	+	BS and CT
11	63	6	Prostatectomy	22.5	−	+	−	CT
12	55	8	Prostatectomy	10.7	−	+	−	CT
13	70	7	Prostatectomy	67	−	−	+	BS and CT
14	72	6	Prostatectomy	15.4	−	−	+ (lung)	Lung Bx
15	64	6	Prostatectomy	3.8	−	−	−	CT and repeat PET
16	68	7	Prostatectomy	14	−	−	+	Bx
17	72	7	Prostatectomy	8.4	−	−	+	CT
18	67	8	Hormone	11.5	−	−	+	BS and CT
19	60	9	Brachytherapy	15.7	−	−	+	BS and CT
20	70	8	Brachytherapy	5.5	−	−	−	CT
21	72	7	Brachytherapy	7.8	+	−	−	Bx
22	77	7	Brachytherapy	4.8	−	−	−	None
23	64	7	Hormone + XRT	41	−	+	−	CT
24	62	7	Hormone + XRT	680	−	+	+	BS and CT
25	57	8	Orchiectomy + XRT	671	+	+	+	BS and CT

FDG, fluorine-18 fluorodeoxyglucose; PET, positron emission tomography; CT, computed tomography; PSA, prostate-specific antigen; BS, bone scan; PLND, pelvic lymph node dissection; Bx, biopsy; XRT, radiation therapy; +, positive; −, negative.
